# Apolipoprotein E epsilon 2 allele and low serum cholesterol as risk factors for gastric cancer in a Chinese Han population

**DOI:** 10.1038/srep19930

**Published:** 2016-01-28

**Authors:** Ranran Kang, Ping Li, Tingting Wang, Xinxiu Li, Zichen Wei, Zhenlian Zhang, Li Zhong, Longlong Cao, Michael G. Heckman, Yun-Wu Zhang, Huaxi Xu, Changming Huang, Guojun Bu, Xiao-Fen Chen

**Affiliations:** 1 Fujian Provincial Key Laboratory of Neurodegenerative Disease and Aging Research, Institute of Neuroscience, Medical College, Xiamen University, Xiamen 361102, China; 2 Department of Gastric Surgery, Fujian Medical University Union Hospital, Fuzhou, Fujian, 350001, China; 3 Division of Biomedical Statistics and Informatics, Mayo Clinic, Jacksonville, FL 32224; 4 Department of Neuroscience, Mayo Clinic, Jacksonville, FL 32224.

## Abstract

Apolipoprotein E (apoE) mediates lipid metabolism both in peripheral and in the brain. The human *APOE* gene has three polymorphic alleles that influence the risk for various types of cancer and neurodegenerative diseases. A potential association between *APOE* allele and the risk for gastric cancer has been implicated, but the specific allele involved and potential associations with the subtype and the grade of cancer malignancy need further clarification. We screened the *APOE* genotype in 550 gastric cancer patients and 550 non-cancer control individuals and found that the presence of the *APOE* ε2 and lower serum total cholesterol are associated with an increased risk for gastric cancer (all P ≤ 0.0005). Interestingly, *APOE* ε2 is also correlated with increased risk for both intestinal and diffuse histotypes but not with TN classification or stage in gastric cancer patients, suggesting that *APOE* polymorphic alleles are associated with the risk of development but unlikely the progression of gastric cancer. Since ε2 carriers have lower levels of serum total cholesterol than non-ε2 carriers, our findings suggest that the increased risk for gastric cancer by *APOE* ε2 allele might be mediated through lowered serum total cholesterol levels.

Gastric cancer is the fourth most commonly diagnosed cancer and the second leading cause of cancer-related death worldwide^1^,^2^. The high mortality associated with this disease might be attributed to the limited understanding on the genetic and environmental risk factors for early diagnosis, prevention and targeted therapy. The alterations of gene expression, such as the improper over-expression of oncogenes or the under-expression or disabling of tumor suppressor genes, have been associated with tumorigenesis^3^. In the study of differential gene expression between gastric cancer and the corresponding normal mucosa, apoE was found to be highly expressed in gastric cancer^4^. In particular, high apoE expression was correlated with deeper tumor invasion or more positive lymph node metastasis, contributing to shorter survival^4^. Therefore, the expression level of apoE may be a potential biomarker for predicting the malignancy of gastric cancer.

ApoE is a 299-amino acid glycoprotein that plays a key role in lipid transport and lipoprotein metabolism by binding to members of the low-density lipoprotein receptor family^5^,^6^,^7^. In addition, apoE has been shown to be involved in several biological events including nerve regeneration, antioxidant activities, immune response, as well as the modulation of tumor cell growth, metastasis induction and angiogenesis^8^,^9^,^10^,^11^. The *APOE* gene consists of four exons and three introns, and is polymorphic on two single nucleotides resulting in three different alleles (ε2, ε3 and ε4) and six *APOE* genotypes (ε2/ε2, ε2/ε3, ε2/ε4, ε3/ε3, ε3/ε4 and ε4/ε4)^12^. The ε2, ε3 and ε4 alleles exhibit different affinity for lipids and receptors; and have a world-wide frequency of 8.4%, 77.9% and 13.7%, respectively^13^,^14^. In addition, the *APOE* genotypes were shown to determine its protein levels in the brain, cerebrospinal fluid, and serum, with ε2/ε2 > ε3/ε3 > ε4/ε4^15^.

*APOE* gene polymorphism leads to an alteration in lipid and lipoprotein metabolism^16^,^17^. In general, compared to the individuals with the ε3 allele, serum levels of total cholesterol, low-density lipoprotein cholesterol (LDL-cholesterol) and apolipoprotein B (apoB) tend to be lower for those with the ε2 allele and higher for ε4 carriers^16^,^18^,^19^,^20^. Correlation between *APOE* gene polymorphism and high-density lipoprotein cholesterol (HDL-cholesterol) level was noted in some studies^16^,^21^, but not in others^20^,^22^. So far, a large number of cross-sectional and prospective studies have reported that low serum cholesterol levels are associated with higher risk for various cancers including gastric cancer^23^,^24^,^25^,^26^.

In our current study, we also observed a strong inverse association between serum total cholesterol levels and the risk for gastric cancer. In addition, we observed significant lower levels of total cholesterol, LDL-cholesterol and apoB in the ε2 carriers which are consistent with previous reports. However, we only observed a trend of higher levels of total cholesterol, LDL-cholesterol in the ε4 carriers. Intriguingly, the presence of the *APOE* ε2 allele is also associated with higher risk for both intestinal and diffuse types of gastric cancer. Since ε2 carriers have lower levels of serum total cholesterol than non-ε2 carriers, our findings suggested that the increased risk for gastric cancer by *APOE* ε2 allele might be mediated through lowered serum total cholesterol levels.

## Results

### Subject description

A summary of the characteristics for the 550 gastric cancer patients and the 550 cancer-free controls is displayed in Table 1. Median age was 63 years (Range: 18–87 years) in gastric cancer patients and 59 years (Range: 22–84 years) in controls. Male gender was most common in both gastric cancer patients (71.5%) and controls (69.1%). Median BMI was 21.5 (Range: 14.5–32.2) in gastric cancer patients and 24.4 (Range: 17.5–59.7) in controls. In gastric cancer patients, T classification was most commonly either T3 (48.8%) or T4 (33.9%); N classification was fairly evenly distributed but most commonly N3 (40.0%); and stage was most commonly III (61.3%). There are 286 patients (52.0%) and 264 patients (48.0%) for intestinal and diffuse gastric cancer subtype, respectively. Serum lipid parameters and the prevalence of *APOE* genotypes for both patients and controls are also summarized in Table 1.

### Comparisons of demographic variables and serum lipid parameters according to the presence or absence of *APOE* ε4 and ε2 in all subjects

Comparisons of demographic variables and serum lipid profile according to the presence or absence of the *APOE* ε4 and ε2 alleles in all subjects are displayed in Table 2. After adjustment for multiple testing (P ≤ 0.005 considered significant), there were differences between subjects with and without a copy of ε4 regarding apoB (Median: 0.99 vs. 0.93, P = 0.0006) and apoA1/apoB ratio (Median: 1.22 vs. 1.31, P = 0.0001). There were significant differences between subjects with and without a copy of ε2 in the overall sample regarding total cholesterol (Median: 4.72 vs. 5.09, P = 0.0006), LDL-cholesterol (Median: 2.94 vs. 3.35, P < 0.0001), apoB (Median: 0.87 vs. 0.96, P < 0.0001), and apoA1/apoB ratio (Median: 1.47 vs. 1.27, P < 0.0001).

### Evaluation of risk factors for gastric cancer

An evaluation of risk factors for gastric cancer is provided in Table 3. In single variable analysis without adjusting for potential confounding variables, there was strong evidence of an association with increased risk of gastric cancer for older age (P < 0.0001) and decreased BMI (P < 0.0001), but no association was evident for gender (P = 0.39). Additionally, risk of gastric cancer was significantly higher for individuals with lower levels of triglycerides (P < 0.0001), total cholesterol (P < 0.0001), HDL-cholesterol (P < 0.0001), LDL-cholesterol (P < 0.0001), apoA1 (P < 0.0001), and apoB (P < 0.0001). There was a trend toward an increased risk of gastric cancer for subjects with a copy of the *APOE* ε2 allele; however, this did not survive correction for multiple testing (P = 0.032). There was no statistically significant evidence of an association with risk of gastric cancer for *APOE* ε4 (P = 0.87) or apoA1/B ratio (P = 0.087).

In multivariable analysis we adjusted for age, BMI, and other variables (see Table 3 legend for details). In this multivariable analysis, there was still strong evidence of an association with increased risk of gastric cancer for age (P < 0.0001), BMI (P < 0.0001), total cholesterol (P < 0.0001), HDL-cholesterol (P < 0.0001), apoA1 (P < 0.0001), and apoA1/B ratio (P < 0.0001). Additionally, the association between *APOE* ε2 and increased risk of gastric cancer strengthened and became significant after adjustment for multiple testing (P = 0.0004). Associations with gastric cancer for triglycerides (P = 0.061), LDL cholesterol (P = 0.35) and apoB (P = 0.48) weakened in multivariable analysis and were no longer even nominally significant, while the lack of association of gender and *APOE* ε4 with risk of gastric cancer remained unchanged (P ≥ 0.30). Intriguingly, when examining the association between *APOE* ε2 and gastric cancer subtype, an increased risk of gastric cancer was noted in ε2 carriers for both intestinal gastric cancer (P = 0.017) and diffuse gastric cancer (P = 0.0006) (Table 4).

### Analysis of the associations of *APOE* genotypes with TN classification and stage in gastric cancer patients

Associations of *APOE* ε4 and ε2 with T classification, N classification, and stage in gastric cancer patients are displayed in Table 5. There was no evidence of an association between ε4 and T classification (P = 0.38), N classification (P = 0.96), or stage (P = 0.59), and also no evidence of an association between ε2 and T classification (P = 0.63), N classification (P = 0.78), or stage (P = 0.23).

## Discussion

Our case-control study of 550 gastric cancer patients and 550 cancer-free controls evaluated for the first time the effects of *APOE* gene polymorphism and serum cholesterol levels on the risk of gastric cancer among a Chinese Han population. Our results show that the presence of *APOE* ε2 allele and low serum levels of total cholesterol, HDL-cholesterol, apoA1 and apoA1/B ratio are associated with an increased risk of gastric cancer. Interestingly, *APOE* ε2 is also correlated with increased risk for both intestinal and diffuse histotypes. However, *APOE* genotypes do not appear to influence the invasion and metastasis of gastric cancer as reflected by TN classification and stage.

In gastric cancer, serum cholesterol levels have been reported to inversely correlate with the risk of disease^25^,^27^,^28^. In our current study, we also observed a strong inverse association between serum total cholesterol levels and the risk of gastric cancer. To date, the underlying mechanism for such an association remains unclear. Previous reports have shown that the serum levels of total cholesterol, LDL-cholesterol and apoB levels are associated with *APOE* genotypes, with individuals carrying the ε2 allele having lower and those carrying the ε4 displaying higher levels compared to the more common ε3 allele^16^,^18^,^19^,^20^. In our studied population, we observed significant lower levels of total cholesterol, LDL-cholesterol and apoB in the ε2 carriers which are consistent with previous reports. Additionally, we observed a significant higher level of apoB in the ε4 carriers. However, we only observed a trend of higher levels of total cholesterol, LDL-cholesterol in the presence of ε4 allele (Table 2). Since ε2 carriers have lower levels of serum total cholesterol than non-ε2 carriers, our findings suggest that the increased risk effect for gastric cancer by *APOE* ε2 allele might be mediated through lowered total cholesterol level.

The effect of *APOE* genotypes on the risk of numerous cancers has been previously investigated, including breast, prostate, ovarian, colorectal, head and neck cancer^29^,^30^,^31^,^32^,^33^,^34^,^35^,^36^. However, the results have been conflicting which might be attributed to limited sample sizes or the inherent differences among different ethnic populations. Our current study in a Chinese Han population observed that the presence of ε2 allele is associated with an increased risk for gastric cancer with OR of 2.34 in multivariable analysis. In contrast, a previous study reported a protective effect of the *APOE* ε2 allele against gastric cancer in an Italian Caucasian population^37^. The discrepancy between these two studies might be attributed to the different ethnic groups that were studied, or potentially due to unmeasured confounding variables. Future extension of these findings is necessary to clarify and further understand the association between *APOE* ε2 and gastric cancer risk in both ethnic populations.

ApoE has recently been identified as a potential tumor-associated marker in gastric cancer from gene expression analysis^4^,^38^,^39^. Higher apoE expression was found in gastric cancer, particularly in advanced T and N grades. We therefore examined the association between *APOE* genotypes and T classification, N classification, and stage in gastric cancer patients. Our data showed no significant association between *APOE* genotypes and various classifications or stage of gastric cancer, suggesting that *APOE* polymorphic alleles are associated with the risk of development but unlikely the progression of gastric cancer.

In summary, our study confirmed the associations of lower levels of serum cholesterol with the incidence of gastric cancer in a Chinese Han population. Importantly, our study reports for the first time that *APOE* ε2 as a risk allele for both intestinal and diffuse types of gastric cancer in our studied population, which might be partly attributed to the lower serum total cholesterol of ε2 carriers compared to those with ε3 or ε4 alleles.

## Methods

Approval for this study was obtained from the Ethics Committees of the Xiamen University and Fujian Medical University Union Hospital. All experiments were performed in accordance with the approved guidelines. All individuals who participated in this study gave written informed consent.

### Study subjects

A total of 550 gastric cancer patients with histological confirmation and 550 cancer-free controls seen at the Fujian Medical University Union Hospital in China between 2011 and 2014 were included in this case–control study. Information was collected regarding age, gender, body mass index (BMI), and serum lipid parameters which include the levels of triglycerides, total cholesterol, HDL-cholesterol, LDL-cholesterol, apoA1, apoB, and apoA1/apoB ratio. None of the patients underwent pre-operative chemotherapy or radiation therapy. Histopathological evaluations were performed with reference to the Japanese Classification of Gastric Carcinoma, 3^rd^ English edition^40^.

### *APOE* genotyping and quality control

Genomic DNA was extracted from peripheral blood using a DNA extraction kit (Zeesan Biotech, Xiamen). Genotyping of the two *APOE* SNPs (rs429358:T/C; rs7412:T/C) was carried out using the *APOE* SNP genotyping kits (Memorigen Biotech, Xiamen, China) and the Applied Biosystems® 7500 real-time PCR Systems (Applied Biosystems, Foster City, CA). Data analysis was performed by measuring the allele-specific fluorescence. As a measure for quality control, three samples with known *APOE* genotypes were included in each assay. Additionally, the genotyping analysis was blinded to the subject’s case or control status. Finally, 10% of the total samples were randomly selected and retested with 100% concordance. There was no evidence of a departure from Hardy-Weinberg Equilibrium in study controls for either rs429358 (P = 0.79) or rs7412 (P = 0.71).

### Statistical analysis

Continuous variables were summarized with the sample median, minimum, 25^th^ percentile, 75^th^ percentile, and maximum. Categorical variables were summarized with number and percentage of patients. Due to the small number of subjects with *APOE* ε4/ε4 and ε2/ε2 genotypes, we utilized a dominant model in analyzing both *APOE* ε4 (presence vs. absence of the ε4 allele) and ε2 (presence vs. absence of the ε2 allele) in all analysis. Comparisons of demographic variables and serum lipid parameters according to the presence of the *APOE* ε4 or ε2 allele were made using a Wilcoxon rank sum test or Fisher’s exact test. Risk factors for gastric cancer were evaluated using single variable (i.e. unadjusted) and multivariable logistic regression models. Multivariable models were adjusted for all variables that were associated with risk of gastric cancer in single variable analysis with a p-value of 0.10 or lower, although there were some exceptions to this due to the high degree of correlation between many of the variables of interest and the resulting potential for collinearity (see Table 3 legend for details). Odds ratios (ORs) and 95% confidence intervals (CIs) were estimated. For easier interpretation of results, all continuous variables were categorized based on approximate sample quartiles for use in association analysis. The association between *APOE* ε2 and gastric cancer subtype was evaluated using single variable (i.e. unadjusted) and multivariable logistic regression models. Multivariable models were adjusted for all variables that were associated with risk of gastric cancer in single variable analysis with a p-value of 0.10 or lower (see Table 4 legend for details). Associations of *APOE* ε4 and ε2 with TN classification and stage in gastric cancer patients were examined using a Wilcoxon rank sum test.

In order to adjust for multiple testing, we utilized a Bonferroni correction separately for each group of similar tests. Specifically, p-values ≤ 0.0050 were considered as significant when comparing demographic variables and serum lipid parameters according to presence of ε4 and ε2; p-values ≤ 0.0042 were considered as significant when evaluating risk factors for gastric cancer; p-values ≤ 0.0167 were considered as significant when evaluating associations of ε4 and ε2 with TN classification and stage in gastric cancer patients; and p-values ≤ 0.025 were considered as significant when evaluating the association between *APOE* ε2 and gastric cancer subtype. All statistical analysis was performed using R Statistical Software (version 2.14.0; R Foundation for Statistical Computing, Vienna, Austria).

## Additional Information

**How to cite this article**: Kang, R. *et al.* Apolipoprotein E epsilon 2 allele and low serum cholesterol as risk factors for gastric cancer in a Chinese Han population. *Sci. Rep.* **6**, 19930; doi: 10.1038/srep19930 (2016).

## Figures and Tables

**Table 1:** Characteristics of the study population.

Variable	Gastric cancer patients (N = 550)	Controls (N = 550)
Age (years)	63 (18, 55, 69, 87)	59 (22, 54, 64, 84)
Gender		
Male	393 (71.5%)	380 (69.1%)
Female	157 (28.5%)	170 (30.9%)
BMI	21.5 (14.5, 19.6, 23.4, 32.2)	24.4 (17.5, 22.6, 26.3, 59.7)
T classification		
T1	20 (3.6%)	N/A
T2	75 (13.7%)	N/A
T3	268 (48.8%)	N/A
T4	186 (33.9%)	N/A
N classification		
N0	120 (21.9%)	N/A
N1	82 (15.0%)	N/A
N2	127 (23.2%)	N/A
N3	219 (40.0%)	N/A
Stage		
I	41 (8.0%)	N/A
II	134 (26.1%)	N/A
III	315 (61.3%)	N/A
IV	24 (4.7%)	N/A
Gastric cancer subtype		
Intestinal	286 (52.0%)	N/A
Diffuse	264 (48.0%)	N/A
*APOE* genotype		
ε2/ε2	3 (0.5%)	1 (0.2%)
ε2/ε3	81 (14.7%)	60 (10.9%)
ε2/ε4	7 (1.3%)	5 (0.9%)
ε3/ε3	371 (67.5%)	396 (72.0%)
ε3/ε4	80 (14.5%)	85 (15.5%)
ε4/ε4	8 (1.5%)	3 (0.5%)
Triglycerides (mmol/L)	1.01 (0.39, 0.79, 1.32, 8.69)	1.18 (0.27, 0.84, 1.63, 6.14)
Total cholesterol (mmol/L)	4.67 (1.80, 3.97, 5.45, 8.85)	5.33 (0.91, 4.63, 6.03, 11.08)
HDL-cholesterol (mmol/L)	1.24 (0.47, 1.00, 1.52, 2.78)	1.41 (0.56, 1.16, 1.74, 3.18)
LDL-cholesterol (mmol/L)	3.02 (0.73, 2.51, 3.76, 7.70)	3.52 (0.36, 2.96, 4.14, 9.14)
ApoA1 (g/L)	1.06 (0.35, 0.82, 1.35, 2.29)	1.34 (0.77, 1.17, 1.48, 4.50)
ApoB (g/L)	0.91 (0.29, 0.75, 1.08, 2.10)	0.97 (0.08, 0.83, 1.11, 1.79)
ApoA1/apoB ratio	1.20 (0.25, 0.90, 1.53, 8.07)	1.37 (0.50, 1.12, 1.76, 5.93)

The sample median (minimum, 25th percentile, 75th percentile, maximum) is given for continuous variables. Information was unavailable regarding T classification (1 gastric cancer patient), N classification (2 gastric cancer patients), stage (36 gastric cancer patients), ApoA1 (1 control), ApoB (1 control), and ApoA1/apoB ratio (1 control).

**Table 2:** Comparison of demographic variables and serum lipid parameters according to the presence or absence of the *APOE* ε4 and ε2 alleles in all subjects.

Variable	Comparisons according to presence of ε4			Comparisons according to presence of ε2		
	ε4 allele present (N = 188)	ε4 allele not present (N = 912)	P-value	ε2 allele present (N = 157)	ε2 allele not present (N = 943)	P-value
Age (years)	60 (25, 84)	60 (18, 87)	0.37	61 (25, 81)	60 (18, 87)	0.046
Gender			1.00			0.35
Male	132 (70.2%)	641 (70.3%)		105 (66.9%)	668 (70.8%)	
Female	56 (29.8%)	271 (29.7%)		52 (33.1%)	275 (29.2%)	
BMI	23.3 (14.7, 38.3)	22.9 (14.5, 59.7)	0.40	23.1 (15.2, 32.4)	22.9 (14.5, 59.7)	0.94
Triglycerides (mmol/L)	1.19 (0.46, 5.10)	1.06 (0.27, 8.69)	0.006	1.11 (0.45, 4.64)	1.08 (0.27, 8.69)	0.62
Total cholesterol (mmol/L)	5.10 (2.29, 10.24)	5.03 (0.91, 11.08)	0.16	4.72 (1.80, 8.22)	5.09 (0.91, 11.08)	0.0006
HDL-cholesterol (mmol/L)	1.27 (0.50, 3.18)	1.34 (0.47, 3.02)	0.034	1.39 (0.47, 2.78)	1.32 (0.48, 3.18)	0.035
LDL-cholesterol (mmol/L)	3.46 (1.24, 7.71)	3.25 (0.36, 9.14)	0.016	2.94 (0.73, 7.70)	3.35 (0.36, 9.14)	< 0.0001
ApoA1 (g/L)	1.18 (0.44, 3.69)	1.24 (0.35, 4.50)	0.020	1.28 (0.35, 2.91)	1.23 (0.40, 4.50)	0.11
ApoB (g/L)	0.99 (0.37, 2.03)	0.93 (0.08, 2.10)	0.0006	0.87 (0.29, 1.97)	0.96 (0.08, 2.10)	< 0.0001
ApoA1/apoB ratio	1.22 (0.39, 3.73)	1.31 (0.25, 8.07)	0.0001	1.47 (0.44, 8.07)	1.27 (0.25, 5.93)	< 0.0001

The sample median (minimum, maximum) is given for continuous variables. P-values result from a Wilcoxon rank sum test or Fisher’s exact test. P-values of 0.0050 or lower were considered as statistically significant after applying a Bonferroni adjustment for multiple testing.

**Table 3:** Evaluation of risk factors for gastric cancer.

Variable	Single variable analysis		Multivariable analysis	
	OR (95% CI)	P-value	OR (95% CI)	P-value
Age^1^	Overall test of difference: P < 0.0001		Overall test of difference: P < 0.0001	
≤55	1.00 (reference)	N/A	1.00 (reference)	N/A
55.01–60	0.56 (0.40, 0.79)	0.001	0.60 (0.38, 0.94)	0.024
60.01–65	1.08 (0.76, 1.53)	0.66	0.88 (0.56, 1.39)	0.58
>65	2.28 (1.66, 3.13)	<0.0001	2.12 (1.40, 3.22)	0.0004
Gender^1^				
Female	1.00 (reference)	N/A	1.00 (reference)	N/A
Male	1.12 (0.86, 1.45)	0.39	1.00 (0.70, 1.42)	>0.99
BMI^1^	Overall test of difference: P < 0.0001		Overall test of difference: P < 0.0001	
>25	1.00 (reference)	N/A	1.00 (reference)	N/A
23.01–25	2.69 (1.84, 3.93)	<0.0001	2.96 (1.87, 4.70)	0.0004
21.01–23	4.82 (3.30, 7.03)	<0.0001	6.69 (4.17, 10.75)	<0.0001
≤21	17.02 (11.26, 25.73)	<0.0001	27.23 (15.96, 46.45)	<0.0001
*APOE* ε4 allele^2^				
Not present	1.00 (reference)	N/A	1.00 (reference)	N/A
Present	1.03 (0.75, 1.40)	0.87	1.25 (0.82, 1.92)	0.30
*APOE* ε2 allele^1^				
Not present	1.00 (reference)	N/A	1.00 (reference)	N/A
Present	1.45 (1.03, 2.05)	0.032	2.34 (1.46, 3.76)	0.0004
Triglycerides^3^	Overall test of difference: P < 0.0001		Overall test of difference: P = 0.061	
>1.50	1.00 (reference)	N/A	1.00 (reference)	N/A
1.091–1.50	1.64 (1.17, 2.31)	0.004	1.74 (1.09, 2.77)	0.020
0.811–1.09	2.02 (1.44, 2.85)	<0.0001	1.65 (1.01, 2.72)	0.046
≤0.81	2.08 (1.48, 2.92)	<0.0001	1.24 (0.71, 2.15)	0.45
Total cholesterol^4^	Overall test of difference: P < 0.0001		Overall test of difference: P < 0.0001	
>5.75	1.00 (reference)	N/A	1.00 (reference)	N/A
5.031–5.75	1.78 (1.26, 2.53)	0.001	1.75 (1.16, 2.65)	0.008
4.291–5.03	2.22 (1.57, 3.14)	<0.0001	1.91 (1.25, 2.92)	0.003
≤4.291	5.97 (4.13, 8.63)	<0.0001	6.28 (3.98, 9.89)	<0.0001
HDL-cholesterol^5^	Overall test of difference: P < 0.0001		Overall test of difference: P < 0.0001	
>1.63	1.00 (reference)	N/A	1.00 (reference)	N/A
1.331–1.63	1.77 (1.25, 2.50)	0.001	2.52 (1.65, 3.88)	<0.0001
1.081–1.33	1.81 (1.28, 2.55)	0.0007	3.08 (1.97, 4.81)	<0.0001
≤1.08	3.34 (2.36, 4.73)	<0.0001	5.99 (3.67, 9.78)	<0.0001
LDL-cholesterol^6^	Overall test of difference: P < 0.0001		Overall test of difference: P = 0.35	
>3.97	1.00 (reference)	N/A	1.00 (reference)	N/A
3.271–3.97	1.37 (0.97, 1.93)	0.072	1.11 (0.71, 1.72)	0.65
2.711–3.27	1.96 (1.39, 2.77)	0.0001	1.15 (0.74, 1.81)	0.53
≤2.71	4.16 (2.92, 5.94)	<0.0001	1.54 (0.94, 2.51)	0.084
ApoA1^7^	Overall test of difference: P < 0.0001		Overall test of difference: P < 0.0001	
>1.45	1.00 (reference)	N/A	1.00 (reference)	N/A
1.231–1.45	0.87 (0.61, 1.24)	0.43	1.03 (0.67, 1.56)	0.90
0.991–1.23	1.44 (1.02, 2.03)	0.037	1.73 (1.12, 2.62)	0.013
≤0.99	10.82 (7.11, 16.47)	<0.0001	13.41 (7.93, 22.67)	<0.0001
ApoB^8^	Overall test of difference: P < 0.0001		Overall test of difference: P = 0.48	
>1.09	1.00 (reference)	N/A	1.00 (reference)	N/A
0.951–1.09	0.83 (0.59, 1.16)	0.27	0.85 (0.54, 1.33)	0.48
0.791–0.95	1.10 (0.79, 1.54)	0.57	0.74 (0.47, 1.16)	0.19
≤0.79	1.83 (1.30, 2.56	0.0005	0.99 (0.61, 1.61)	0.97
ApoA1/apoB ratio^9^	Overall test of difference: P = 0.087		Overall test of difference: P < 0.0001	
>1.65	1.00 (reference)	N/A	1.00 (reference)	N/A
1.291–1.65	1.35 (0.96, 1.90)	0.082	2.89 (1.87, 4.47)	<0.0001
1.041–1.29	1.49 (1.06, 2.10)	0.023	4.60 (2.86, 7.42)	<0.0001
≤1.04	3.73 (2.62, 5.30)	<0.0001	11.84 (7.18, 19.52)	<0.0001

OR = odds ratio; CI = confidence interval. ORs, 95% CIs, and p-values result from logistic regression models. Multivariable models were adjusted for age and BMI, as well as other variables that were associated with risk of gastric cancer in single variable analysis with a p-value of 0.10 or lower. Because many of these other variables were very highly correlated with one another which results in high potential for collinearity, all variables satisfying this criteria could not always be adjusted for in all models, and therefore model adjustments in multivariable analysis were as follows:

^1^Adjusted for age, BMI, *APOE* ε2, triglycerides, total cholesterol, HDL-cholesterol, LDL-cholesterol, ApoA1, ApoB, and ApoA1/apoB ratio.

^2^Adjusted for age, BMI, *APOE* ε4, triglycerides, total cholesterol, HDL-cholesterol, LDL-cholesterol, ApoA1, ApoB, and ApoA1/apoB ratio.

^3^Adjusted for age, BMI, *APOE* ε2, triglycerides, HDL-cholesterol, LDL-cholesterol, ApoA1, ApoB, and ApoA1/apoB ratio.

^4^Adjusted for age, BMI, *APOE* ε2, total cholesterol, and ApoA1/apoB ratio.

^5^Adjusted for age, BMI, *APOE* ε2, triglycerides, HDL-cholesterol, LDL-cholesterol, and ApoB.

^6^Adjusted for age, BMI, *APOE* ε2, triglycerides, LDL-cholesterol, ApoA1, and ApoB.

^7^Adjusted for age, BMI, *APOE* ε2, triglycerides, HDL-cholesterol, LDL-cholesterol, and ApoA1.

^8^Adjusted for age, BMI, *APOE* ε2, triglycerides, HDL-cholesterol, ApoA1, and ApoB.

^9^Adjusted for age, BMI, *APOE* ε2, triglycerides, and total cholesterol. P-values of 0.0042 or lower were considered as statistically significant after applying a Bonferroni adjustment for multiple testing.

**Table 4:** Association between *APOE* ε2 and gastric cancer subtype.

Gastric cancer subtype	Single variable analysis		Multivariable analysis	
	OR (95% CI)	P-value	OR (95% CI)	P-value
Intestinal	1.41 (0.94, 2.11)	0.10	2.01 (1.13, 3.56)	0.017
Diffuse	1.51 (1.00, 2.27)	0.051	2.86 (1.57, 5.21)	0.0006

OR = odds ratio; CI = confidence interval. ORs, 95% CIs, and p-values result from logistic regression models. Multivariable models were adjusted for all variables that were associated with risk of overall gastric cancer in single variable analysis with a p-value of 0.10 or lower, which were age, BMI, triglycerides, total cholesterol, HDL-cholesterol, LDL-cholesterol, ApoA1, ApoB, and ApoA1/apoB ratio.

**Table 5:** Association of *APOE* ε4 and ε2 with TN classification and stage in gastric cancer patients.

Variable	Comparison of TN classification and stage according to presence of the *APOE*ε4 allele			Comparison of TN classification and stage according to presence of the *APOE*ε2 allele		
	ε4 allele present (N = 95)	ε4 allele not present (N = 455)	P-value	ε2 allele present (N = 91)	ε2 allele not present (N = 459)	P-value
T classification						
T1	3 (3.2%)	17 (3.7%)	0.38	5 (5.6%)	15 (3.3%)	0.63
T2	12 (12.6%)	63 (13.9%)		9 (10.0%)	66 (14.4%)	
T3	54 (56.8%)	214 (47.1%)		49 (54.4%)	219 (47.7%)	
T4	26 (27.4%)	160 (35.2%)		27 (30.0%)	159 (34.6%)	
N classification						
N0	23 (24.2%)	97 (21.4%)	0.96	17 (18.9%)	103 (22.5%)	0.78
N1	11 (11.6%)	71 (15.7%)		19 (21.1%)	63 (13.8%)	
N2	22 (23.2%)	105 (23.2%)		20 (22.2%)	107 (23.4%)	
N3	39 (41.1%)	180 (39.7%)		34 (37.8%)	185 (40.4%)	
Stage						
I	8 (9.0%)	33 (7.8%)	0.59	6 (7.3%)	35 (8.1%)	0.23
II	23 (25.8%)	111 (26.1%)		25 (30.5%)	109 (25.2%)	
III	56 (62.9%)	259 (60.9%)		51 (62.2%)	264 (61.1%)	
IV	2 (2.2%)	22 (5.2%)		0 (0.0%)	24 (5.6%)	

P-values result from a Wilcoxon rank sum test. Information was unavailable regarding T classification (1 gastric cancer patient), N classification (2 gastric cancer patients), and stage (36 gastric cancer patients). P-values of 0.0167 or lower were considered as statistically significant after applying a Bonferroni adjustment for multiple testing.
